# Antibiotic Prophylaxis in Laparoscopic Cholecystectomy: A Randomized Controlled Trial

**DOI:** 10.1371/journal.pone.0106702

**Published:** 2014-09-05

**Authors:** Yoichi Matsui, Sohei Satoi, Masaki Kaibori, Hideyoshi Toyokawa, Hiroaki Yanagimoto, Kosuke Matsui, Morihiko Ishizaki, A-Hon Kwon

**Affiliations:** Department of Surgery, Kansai Medical University, Hirakata, Osaka, Japan; Copenhagen University Hospital Gentofte, Denmark

## Abstract

**Background:**

Recent meta-analyses concluded that antibiotic prophylaxis is not warranted in low-risk laparoscopic cholecystectomy. However, most trials in the meta-analyses had a relatively small sample size and were statistically underpowered. In addition, many of the trials mentioned potential cost savings owing to the elimination of prophylactic antibiotics. However, no trial has statistically estimated the cost effectiveness. To evaluate the results of meta-analyses, we conducted a randomized controlled trial on the role of prophylactic antibiotics in low-risk laparoscopic cholecystectomy with an adequate sample size.

**Methods:**

From March 2007 to May 2013, at the Department of Surgery, Kansai Medical University, patients who were scheduled for elective laparoscopic cholecystectomy were randomly assigned to one of two arms: those who were and were not administered prophylactic antibiotics. The primary endpoint was the occurrence of postoperative infections and secondary endpoints were postoperative hospital stay and medical costs.

**Findings:**

During the study period, 518 patients were assigned to the Antibiotics group and 519 to the No antibiotics group. Occurrences of surgical site infections, distant infections and overall infections were significantly lower in the Antibiotics group than in the No antibiotics group (0.8 vs. 3.7%, p = 0.001, OR: 0.205 (95%CI: 0.069 to 0.606); 0.4 vs. 3.1%, p = 0.0004, OR: 0.122 (95%CI: 0.028 to 0.533); 1.2 vs. 6.7%; p<0.0001, OR: 0.162 (95%CI: 0.068 to 0.389), respectively). The postoperative hospital stay was significantly shorter in the Antibiotics group (mean, SD: 3.69±1.56 vs. 4.07±3.00; p = 0.01) and the postoperative medical costs were significantly lower in the Antibiotics group (mean, SD: $766±341 vs. 832±670; p = 0.047). Multivariable analysis showed that independent risk factors for postoperative infectious complications were no prophylactic antibiotics (p<0.0001) and age 65 or older (p = 0.006).

**Conclusions:**

Perioperative administration of prophylactic antibiotics should be recommended in laparoscopic cholecystectomy to prevent postoperative infectious complications and to reduce medical costs.

**Trial Registration:**

UMIN Clinical Trials Registry UMIN000003749.

## Introduction

Administration of prophylactic antibiotics has been recommended by the Centers for Disease Control and Prevention and widely used in clean-contaminated surgery such as cholecystectomy to reduce surgical site infections (SSI). In contrast, several meta-analyses have recently concluded that antibiotic prophylaxis is not warranted in low-risk patients undergoing laparoscopic cholecystectomy.

At present, there are six meta-analyses [1]–[6] that included a total of 20 randomized controlled trials that evaluated the role of prophylactic antibiotics for low-risk laparoscopic cholecystectomy (Table 1) [7]–[26]. All of these randomized studies and their meta-analyses showed no significant differences in the occurrence of postoperative infectious complications between the prophylactic antibiotics group and no prophylaxis group. Thus, they all concluded that prophylactic antibiotics are not needed or warranted for low-risk laparoscopic cholecystectomy. Consequently, a current report documented a trend of not using antibiotic prophylaxis in laparoscopic cholecystectomy [27]. However, most trials in these meta-analyses had a relatively small sample size and were considered to be statistically underpowered for the rare event of infections [28], [29]. A recent comment has highlighted a problem with meta-analyses that reviewed randomized trials with a small sample size in that the true occurrence of postoperative infections might be underestimated [30]. Indeed, several trials included in these meta-analyses also pointed out that a larger sample size would be necessary to detect significant differences because of the rarity of complications [9], [10], [24], [26]. In addition, many of the trials mentioned potential cost savings owing to the elimination of prophylactic antibiotics [8], [10], [13], [14], [18], [21], [25], [26]. However, no trial has statistically estimated the cost effectiveness of eliminating prophylactic antibiotics.

**Table 1:** Meta-analyses regarding prophylactic antibiotics for laparoscopic cholecystectomy.

			Postoperative infection rate					
Published Date (Ref No.)	No. of RCTs§	Total No. Patients (range)		Antibiotics group	No antibiotics group	Odds ratio (95%CI#)	p	Conclusion
2003	5	899	Wound infections	1.5%	2.2%	0.68 (0.24–1.91)	ns*	Do not support the use
(1)		(53–412)	Major infections	0.2%	0.3%	0.93 (0.06–14.9)		of prophylactic
			Distant infections	0.8%	1.6%	0.50 (0.14–1.78)		antibiotics
2004	6	974	Surgical site infections	2.1%	2.9%	0.82 (0.36–1.86)	ns	No need to administer
(2)		(53–412)	Other site infections	0.7%	1.5%	0.82 (0.18–1.90)		routine antibiotics
			Overall infections	2.8%	4.4%	0.69 (0.34–1.43)		
2008	9	1437	Wound infections	1.6%	2.3%	0.71 (0.34–1.48)	ns	Antibiotics do not
(3)		(84–412)	Major infections	0.3%	0.4%	1.03 (0.25–4.20)		prevent infections
			Distant infections	0.8%	2.0%	0.49 (0.13–1.81)		
			Overall infections	2.4%	3.6%	0.66 (0.35–1.24)		
2009	15	2961	Wound infections	1.5%	1.8%	0.79 (0.44–1.41)	ns	Antibiotics are
(4)		(53–635)	Major infections	0.2%	0.1%	2.51 (0.35–17.8)		unnecessary
			Distant infections	0.4%	0.6%	0.53 (0.19–1.50)		
			Overall infections	2.1%	2.5%	0.77 (0.47–1.27)		
2010	11	1664	Surgical site infections	2.7%	3.3%	0.87 (0.49–1.54)	ns	No evidence to support
(5)		(76–412)	Extra-abdominal infections	1.0%	1.8%	0.66 (0.25–1.74)		or refute antibiotics
2011	12	1937	Wound infections	2.3%	2.2%	1.07 (0.59–1.94)	ns	Antibiotics are not
(6)		(53–412)	Major infections	0.4%	0.0%	2.88 (0.3–28.09)		necessary
			Distant infections	1.4%	1.5%	1.01 (0.43–2.36)		
			Overall infections	3.6%	3.4%	1.11 (0.68–1.82)		

§ , randomised controlled trials;

# , confidence interval;

*, not significant.

To confirm the results of the meta-analyses and to determine whether or not cost savings are associated with not using perioperative antibiotics, we conducted a randomized controlled trial that assessed the role of prophylactic antibiotics in postoperative infectious complications and the cost effectiveness of their use in elective low-risk laparoscopic cholecystectomy with a statistically adequate sample size.

## Methods

This randomized trial was conducted at the Department of Surgery, Kansai Medical University. The period of recuruitment was from March 1, 2007 to May 31, 2013, and the last follow-up date was June 30, 2013. The protocol was approved by The Institutional Review Board for Clinical Research of Kansai Medical University Hirakata Hospital (approval No. H070402) before enrollment of participants had began, and written informed consent was obtained from all participating patients. This trial did not achieve the target sample size (1006 cases) until the planed date of recruitment closure (April 30, 2011). Thus, the trial period was extended until May 31, 2013. This extension was also approved by the institutional review board prior to the extension. This study was registered with the University Hospital Medical Information Network-Clinical Trials Registry (UMIN-CTR), registry ID: UMIN000003749 after enrollment of participants had begun. The reason for the delay in registration was that the clinical trial registration system did not have widespread adoption in Japan when we began the trial (2007). When the UMIN-CTR was implemented fully, we registered our trial.

The authors confirm that all ongoing and related trials for this intervention are registered. The protocol for this trial and supporting CONSORT checklist are available as supporting information; see Checklist S1 and Protocol S1.

### Randomization

Patients with gallbladder stones or polyps scheduled to undergo elective laparoscopic cholecystectomy were eligible for enrollment in the study. For statistical analysis, it was estimated that it would be necessary to enroll 503 patients per arm by the end of the study. Enrollment was discontinued at the end of May 2013 because more than 503 cases were enrolled in each arm. Excluded from the study were patients who underwent emergency operations, concurrently underwent another surgery, used insulin or steroids regularly, had a history of allergy to antibiotics, were on haemodialysis, had taken antibiotics within 7 days prior to surgery, were younger than 18 years of age, had severe comorbidities such as Child C liver cirrhosis, or were undergoing chemotherapy for malignancies.

Patients were randomized into either of two groups using a computer-generated random number just before the operation by a third party who telephoned the allocation to the operating theatre. Patients in the Antibiotics group were given a total of three 1-g doses of intravenous cefazolin sodium: the first, just before skin incision, and the second and the third at 12 h and 24 h, respectively, after the operation. If an operation took over 3 h, a further dose of antibiotics was given intravenously. Patients in the No antibiotics group received no antibiotics. The surgeons who provided the assigned treatment and interventions were not masked to the patient's group assignment.

### Endpoints

The primary endpoint was a postoperative infectious complication, including a SSI or distant site infection. Secondary endpoints were length of postoperative hospital stay and postoperative medical costs, including hospitalisation and outpatient costs.

### Surgical Procedures

Conventional laparoscopic cholecystectomy was performed for most of the patients, while the single incision technique was introduced in November 2009 and performed in some of the patients. The standard skin preparation was 10% povidone-iodine solution. Gallbladder was extracted in a plastic bag through an opening made by a 12-mm trocar. The incision at the 12-mm trocar site was closed with 3–0 absorbable sutures, and the other incisions were closed with a skin stapler. Drains were inserted only if there had been gallbladder rupture with bile and/or stone spillage into the abdominal cavity. A balloon catheter was placed into the urinary bladder at the start of surgery in all patients and was removed in the evening on the day of the operation or in the morning of postoperative day 1 at the patient's request. All operations were performed in a single surgical unit by an experienced surgeon [YM] with surgeons in training.

### Outcome Measures

Demographic data, including age and sex, and information on diabetes status, diagnosis (gallstone or others), history of upper abdominal surgery, operative time, and bile spillage during the operation were collected for all patients. Examination for SSIs and other infectious diseases were made until hospital discharge and then again at the first postoperative visit. All patients were followed up for at least 8 days after surgery at the outpatient department. Postoperative hospital stay and postoperative medical costs, including costs for prophylactic or therapeutic antibiotics, postoperative hospital charges, and costs for other extra procedures at the outpatient department, were recorded. Any adverse events that occurred in this trial were assessed according to the Clavien-Dindo Classification and the Common Terminology Criteria for Adverse Events (CTCAE, ver. 4). Any event at Grade 2 or higher was included as an adverse event in this study.

Infectious complications were defined as follows: wound infection, pus discharge from the surgical wound requiring open drainage; major SSIs, intra-abdominal abscesses requiring drainage under ultrasonographic control; and distant infection, any infection remote from the surgical site such as the urinary or respiratory tract requiring consultation with a medical specialist. When patients complained of any urinary symptoms, the diagnosis of a urinary tract infection was made by a urine test that microscopically revealed bacteria in the urine. A doctor in charge of the outpatient department who was unaware of the randomization checked the patients' status at least once on postoperative day 8. According to our institutional guidelines patients with a body temperature above 38°C are considered as being at risk of a potential infectious disease. Therefore, fever of unknown origin (FUO) was declared when a body temperature higher than 38°C was observed at least once on postoperative day 1 and/or day 2 without any other clinical signs of infection. Body temperature was recorded 3 times a day at 8-hour intervals during the entire hospitalization. The fever recorded was not an endpoint.

When a procedure was converted to an open surgery, intravenously administered prophylactic antibiotics were provided at the same time as the open procedure. Extra antibiotics were given to patients in the No antibiotics group and additional antibiotics were given to patients in the Antibiotics group on postoperative day 1 and day 2. These patients were excluded from per-protocol analysis but were included in the intention-to-treat analysis.

If an infectious complication was postoperatively encountered in either group, extra or additional antibiotics were given immediately until there was no evidence of infection. When a fever higher than 38°C was found on postoperative day 1 and/or day 2 (FUO), extra or additional antibiotics were given in either group even if no other infection-related sign was clinically evident. If patients had no postoperative complications during their hospitalizations, the decision of the discharge date was made primarily by the patient.

Postoperative medical costs per patient were calculated according to the National Health Insurance System. The total postoperative cost per patient was obtained by adding up hospital charges, antibiotics cost, and outpatient department costs. Postoperative hospital charges were $200.5 per day, which includes the cost of all medical procedures during the hospitalisation. The fee for 1 g of cefazolin sodium was $7.97. The cost of surgical drainage for an infected wound at the outpatient department was $47, and other expenses for procedures or prescriptions including antibiotics for minor infectious diseases at the outpatient department were according to the National Health Insurance System.

### Statistical Analysis

We expected that the overall infectious complication rate in the Antibiotics group would be 2% and that in the No antibiotics group would be 6%. This trial was designed to detect a 4% difference in the occurrence of postoperative infectious complications between the two groups with a power of 90%. We expected a 6% rate for postoperative infectious complications in the no antibiotics group through our experience with the rate of postoperative infectious complications during the 2 years before starting the trial. The 2% rate was expected according to the rate from the past meta-analyses as shown in Table 1. A sample size of 503 was required in each arm. Statistical methods and data analysis were performed using JMP ver. 10.0.2 (SAS Institute Inc., Cary, NC, USA). The chi-square test was used to analyse categorised variables, whereas the Fisher's exact test was indicated when the expected frequency in a cell is less than 5. The two-tailed unpaired t-test was used to analyse continuous variables. Multivariable logistic regression analysis was carried out using a likelihood ratio test with a nominal scale to find an independent relationship between infectious complications and the risk factors listed in Table 2 (age, sex, diabetes status, diagnosis of gallstone or others, history of upper abdominal surgery, operative time, bile spillage during the operation) and antibiotic prophylaxis. Significance was defined as p<0.05.

**Table 2:** Clinical background and perioperative data.

	Antibiotics group (n = 518)	No antibiotics group (n = 519)
Age 65 or older (yes/no)	197/321	202/317
Gender (male/female)	261/257	229/290
Diabetes mellitus (yes/no)	31/487	35/484
Diagnosis of gallstone (yes/no)	451/67	461/58
History of upper abdominal surgery (yes/no)	24/494	20/499
Operation time >60 min (yes/no)	292/226	281/238
Bile spillage (yes/no)	113/405	104/415

## Results

**Figure 1:**
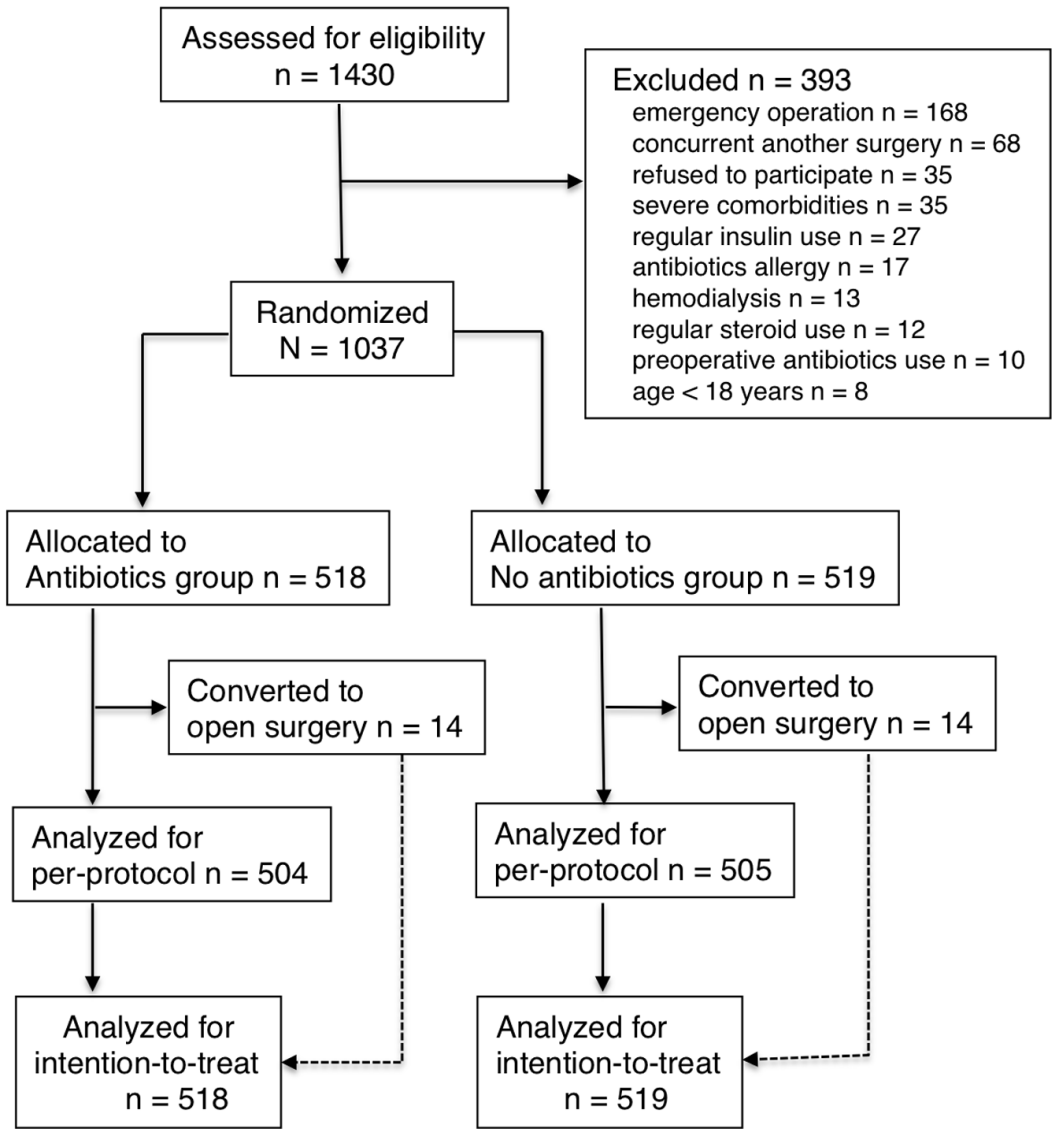
Diagram for the trial.

During the study period, a total of 1430 consecutive patients underwent laparoscopic cholecystectomy at our institution. Of those 1430 patients, 393 were excluded as study participants according to the exclusion criteria. The remaining 1037 patients were randomized into either the Antibiotics group (518 patients) or the No antibiotics group (519 patients) (Fig. 1). Clinical background and perioperative data were shown to be homogeneous between the two groups (Table 2). Follow-up at our outpatient department was complete with the exception of 1 patient in the Antibiotics group and 2 patients in the No antibiotics group who did not visit the hospital after discharge. By phone two weeks after discharge, these three patients were confirmed not to have any difficulty related to their operation.

### Primary endpoint

Occurrence of complications associated with postoperative infections was compared between the two groups (Tables 3 and 4). Per-protocol analysis indicated that the occurrence of both SSIs and distant infections was significantly lower in the Antibiotics group than in the No antibiotics group, resulting in a significantly lower occurrence of overall infectious complications in the Antibiotics group (Table 3). The significant difference in SSIs was mainly caused by the lower rate of wound infections in the Antibiotics group, while the significant difference in distant infections was because there were no urinary tract infections in the Antibiotics group. Results of intention-to-treat analysis were similar to results of per-protocol analysis (Table 4). Three major SSIs in the No antibiotics group were subhepatic abscesses that required drainage percutaneously under ultrasonographic control. As to distant infections other than urinary tract infections, one patient had colitis and another had a respiratory infection in the Antibiotics group while one and two patients, respectively, in the No antibiotics group had colitis and a respiratory infection.

**Table 3:** Occurrence of postoperative infectious complications (primary endpoint per-protocol analysis).

Infectious complications	Antibiotics group (n = 504)	No antibiotics group (n = 505)	p	Odds ratio (95%CI#)
Surgical site infections	4 (0.8%)	14 (2.8%)	0.015	0.281 (0.092–0.858)
Wound infections	4 (0.8%)	13 (2.6%)	0.024	0.303 (0.098–0.935)
Subhepatic abscess	0 (0.0%)	1 (0.2%)	1.000*	-
Distant infections	1 (0.2%)	16 (3.2%)	<0.0001	0.061 (0.008–0.460)
Urinary infections	0 (0.0%)	13 (2.6%)	<0.0001	-
Other infections	1 (0.2%)	3 (0.6%)	0.624*	0.333 (0.034–3.21)
Overall infections	5 (1.0%)	30 (5.9%)	<0.0001	0.159 (0.061–0.412)

# , confidence interval;

*, Fisher's exact test was used.

**Table 4:** Occurrence of postoperative infectious complications (primary endpoint intention-to-treat analysis).

Infectious complications	Antibiotics group (n = 518)	No antibiotics group (n = 519)	p	Odds ratio (95%CI#)
Surgical site infections	4 (0.8%)	19 (3.7%)	0.001	0.205 (0.069–0.606)
Wound infections	4 (0.8%)	16 (3.1%)	0.005	0.245 (0.081–0.737)
Subhepatic abscess	0 (0.0%)	3 (0.6%)	0.249*	-
Distant infections	2 (0.4%)	16 (3.1%)	0.0004	0.122 (0.028–0.533)
Urinary infections	0 (0.0%)	13 (2.5%)	<0.0001	-
Other infections	2 (0.4%)	3 (0.6%)	1.000*	0.667 (0.111–4.01)
Overall infections	6 (1.2%)	35 (6.7%)	<0.0001	0.162 (0.068–0.389)

# , confidence interval;

*, Fisher's exact test was used.

All of the wound infections, urinary tract infections, and respiratory infections had developed after discharge. Therefore, these patients were treated at the outpatient clinic. In the case of wound infections, the doctor in charge of the outpatient department opened and drained the wound in the outpatient department. When the urine test performed for the patients with the complaint of urinary symptoms revealed bacteria microscopically, a doctor in charge diagnosed this as urinary tract infections and began treatment. Two of these patients were diagnosed with acute prostatitis, and one of the two was admitted to the Department of Urology for treatment. The remaining patients with urinary problems were diagnosed as having urinary tract infections and treated with antibiotics. The three subhepatic abscesses and the two cases of colitis had developed during hospitalisation; therefore, these patients were treated in hospital.

### Secondary endpoints

Table 5 shows a comparison of the postoperative hospital stay and medical costs between the two groups. The results of the per-protocol analysis showed that the cost for antibiotics per patient was significantly lower in the No antibiotics group since these patients only received antibiotics in response to infectious complications. However, the total medical cost was not significantly different between groups and tended to be higher in the No antibiotics group. Moreover, results from the intention-to-treat analysis showed that the total medical cost per patient was significantly higher in the No antibiotics group. The postoperative hospital stay was significantly longer in the No antibiotics group than in the Antibiotics group.

**Table 5:** Postoperative hospital stay and medical costs (secondary endpoints).

	Antibiotics group (mean ±SD)	No antibiotics group (mean ±SD)	p
Per-protocol analysis			
Hospital stay (day)	3.55±1.21	3.81±1.83	0.009
Cost for antibiotics ($/patient)	24.1±3.4	5.8±26.6	<0.0001
Total medical costs ($/patient)	737.5±243.7	772.7±382.1	0.082
Intention-to-treat analysis			
Hospital stay (day)	3.69±1.56	4.07±3.00	0.010
Cost for antibiotics ($/patient)	24.9±9.5	8.1±32.5	<0.0001
Total medical costs ($/patient)	766.1±340.9	831.9±670.0	0.047

To apply the results to the day surgery situation, the postoperative medical costs were re-calculated excluding the hospital charges in each group. Only the hospital charges for the patients with a major complication or those for the patients converted to an open surgery were added to the medical costs. As a result, the difference in the postoperative costs between the two groups was more remarkable. The postoperative medical costs were also significantly lower in the Antibiotics group than in the No antibiotics group (mean, SE: $73±15 vs. 119±31; p<0.0001, Wilcoxon test).

### Multivariable analysis

Multivariable logistic regression analysis was performed to determine whether the non-use of antibiotics and whether any of the other suspected risk factors listed in Table 2 were independent factors related to postoperative infectious complications. As shown in Table 6, no administration of prophylactic antibiotics and age 65 or older were significant risk factors associated with the overall infectious complications.

**Table 6:** Multivariable logistic regression analysis for overall infectious complications.

	Odds Ratio (95%CI#)	p
No antibiotics administration	6.26 (2.79–16.7)	<0.0001
Age 65 or older	2.52 (1.31–4.98)	0.006
Gender male	1.14 (0.59–2.19)	0.697
Diabetes mellitus	1.04 (0.24–3.10)	0.946
Gallstone	1.26 (0.43–5.40)	0.700
History of upper abdominal surgery	1.38 (0.31–4.47)	0.635
Operation time >60 min	1.16 (0.59–2.32)	0.664
Bile spillage	1.09 (0.48–2.29)	0.832

# , confidence interval.

The relationship between the SSIs and bile spillage was estimated in each group. As a result, the occurrence rate of the SSIs in patients with bile spillage was significantly higher than that in patients with no bile spillage in the No antibiotics group (7.7% with bile spillage vs. 2.7% with no bile spillage, p = 0.025, using the chi-square test), whereas there was no significant difference in the occurrence rate of the SSIs between patients with bile spillage and patients with no bile spillage in the Antibiotic group (0.9% with bile spillage vs. 0.7% with no bile spillage, p = 0.879).

The occurrence of postoperative FUO was significantly lower in the Antibiotics group (0.6% vs. 2.1%, p = 0.027, OR: 0.269, 95%CI: 0.075–0.970).

The adverse events of postoperative complications other than infectious diseases occurred in 12 patients (2.32%) in the Antibiotics group and in 12 patients (2.31%) in the No antibiotics group. Adverse events regarding antibiotics administration occurred in 2 patients (0.39%) in the Antibiotics group and 0 patients (0.00%) in the No antibiotics group. Two patients in the Antibiotics group probably developed allergic reactions just after starting the injection of cefazolin sodium. Eruptions appeared on the body of one patient and blood pressure decreased in the other patient. Administration of antibiotics was immediately discontinued by the attendant anesthesiologist because the anesthesiologist judged these events as possible allergic reactions. These patients immediately recovered from the possible allergic reactions and underwent the laparoscopic cholecystectomy as planned. Both were discharged on day 3 after the surgery. Although neither patient was given any other antibiotics, they were included as patients in the Antibiotics group. There was no mortality among the enrolled patients.

All data of this trial can be accessed in the supporting information.

## Discussion

Past randomized trials and their meta-analyses concluded that laparoscopic cholecystectomy does not require antibiotic prophylaxis because of its association with a low infection rate. In contrast, the present results showed a reduction in infectious complications with three perioperative doses of prophylactic antibiotics in elective low-risk laparoscopic cholecystectomy. Moreover, our results revealed that eliminating the use of prophylactic antibiotics did not result in a cost reduction.

It can be questioned why our results showed that the occurrence of infectious complications was significantly higher in the No antibiotics group than in the Antibiotics group, contrary to results of past trials. There are three possible reasons.

First, most of the previous trials were statistically underpowered due to their relatively small sample sizes. If postoperative infection rates were less than 7%, as in our study, these trials might not be able to detect significant differences with those sample sizes.

Second, approximately half of the trials used only a single dose of antibiotics [9], [10], [13], [14], [21], [22], [24]–[26]. Since most of the trials used only a single dose or a double dose and found no clear cut benefit, we felt that three perioperative doses would be more effective than a single or a double dose. Considering the results of this study, it appears that three doses would be needed to prevent postoperative infections. Even if a cholecystectomy were performed on a day surgery basis, a tablet antibiotic could be administered after day surgery in addition to the single intravenous administration at the time of skin incision.

Third, most of the complications occurred after discharge because the majority of the patients were discharged within a few of days after surgery. Therefore, complications such as wound infections or urinary tract infections might be missed if all patients were not carefully followed up at outpatient clinic. To prevent overlooking these complications, a perfect follow-up rate is needed. In this study, all of the enrolled patients were completely followed up and all complications were detected. Follow-up rates should be an important issue for randomized trials. However, only one trial [14] reported follow-up rates. The perfect follow-up rate among our patients might have resulted in the relatively high occurrence of infectious complications in the No antibiotics group compared to findings of past trials.

The second and the third reasons described above might explain why the meta-analyses did not find significant differences even though sufficient sample sizes were achieved in the meta-analyses by pooling the samples from each trial. Issues of sample size, dose of antibiotics, and follow-up rates might explain the contradiction of our results with past results. In fact, 5 of the 6 meta-analyses showed a tendency of higher rates of infectious complications in the No antibiotics arm (Table 1). This tendency would support our outcomes.

Another issue is why medical costs were unexpectedly higher in the No antibiotics group. It is generally thought that omitting prophylactic antibiotics would lower medical costs. Most of the previous trials mentioned potential cost savings with elimination of antibiotic prophylaxis [8], [10], [13], [14], [18], [21], [25], [26]. However, no trials statistically estimated cost effectiveness. The present study for the first time examined cost effectiveness with regard to perioperative antibiotics administration and obtained unexpected results. There are three possible reasons for our findings.

First, in the No antibiotics group, three patients developed severe infectious complications, that is, subhepatic abscesses, and required prolonged hospitalisation averaging 28 days. These prolonged hospital stays were the main contributors to the total medical costs. Hospital charges for prolonged hospital stays easily overcame savings from omitting the use of perioperative antibiotics in the No antibiotics group. The average hospital charge for these three patients was $5,614 per patient. This average charge per patient is equivalent to the total savings for 235 patients who are spared prophylactic antibiotics. Costs for patients with these severe complications wiped out the savings from the non-use of perioperative antibiotics because the widely used prophylactic antibiotics are inexpensive. In this study, the cost for prophylactic antibiotics per patient was only $23.9 for 3 g of cefazolin.

Second, the higher occurrence of FUO in the No antibiotics group compared with the Antibiotics group was a factor in the longer hospitalisations and extra administration of antibiotics in the former group, which elevated medical costs in the No antibiotics group. Although the causes of FUO were unclear, the lower rate of FUO in the Antibiotics group suggested that the antibiotics had some effects in preventing minor systemic infections that developed without any other clinical signs of infection. Severe infectious complications and FUOs could also account for the longer hospital stay in the No antibiotics group.

Third, the medical costs related to outpatient department treatment were higher in the No antibiotics group because of the higher occurrence of complications occurring after discharge in comparison with the Antibiotics group. The outpatient department needed to open and drain wounds, make referrals to the Urology Department, and administer therapeutic antibiotics, which had some role in the higher medical costs in the No antibiotics group compared with the Antibiotics group.

These infection-related complications possibly elevated the total amount of postoperative medical costs so that the cost savings through elimination of perioperative antibiotics were negated. We found no cost-saving effects with elimination of antibiotics in the per-protocol analysis. Moreover, intention-to-treat analysis showed that medical costs were unexpectedly higher in the No antibiotics group. Considering cost effectiveness, prophylactic antibiotics would be worth administering even in patients undergoing low-risk laparoscopic cholecystectomy.

The 24-h schedule of three doses of intravenous antibiotics for elective laparoscopic cholecystectomy poses problems and difficulties with day case surgery, which is a common practice, particularly in the Western world. Since patients would not be available for intravenous antibiotics, the prophylactic antibiotics could be administered orally after day surgery. There is little reason that the use of day surgery would reduce postoperative complications; therefore, the occurrence of postoperative infections after a day surgery would be similar to the occurrence after inpatient surgery. Although the day-case process would reduce equally the total medical costs of the two arms, the difference in medical costs between the two arms would not change. Consequently, the statistical differences in costs might become more remarkable. Actually, as shown in the Results section, the postoperative costs excluded the hospital charges showed statistically more remarkable difference between the two groups. Therefore, even in day surgery cases, prophylactic antibiotics using tablets after day case operations may also reduce medical costs through preventing postoperative infections. Thus, our results for patients who were hospitalized for a few days after surgery would not be inconsistent with the outcome in the day surgery situation.

In this study, there was a significantly higher occurrence of urinary tract infections in the No antibiotics group than in the Antibiotics group. The balloon catheter placed into the urinary bladder at the operation probably caused such infections. The urinary complications might have been prevented if the catheters were not placed, even in the No antibiotics group. If urinary infections are excluded from our data analyses, the difference in overall infection rates would be reduced, whereas the difference of SSI rates does not change. The ethics of not administering antibiotic prophylaxis at the time of removal of short-term urinary catheters is questionable. A meta-analysis showed that removal of short-term urinary catheters under antibiotic coverage significantly reduced the risk of urinary tract infection [31].

The multivariable analysis indicated that independent risk factors for the overall infectious complications were not only lack of prophylactic antibiotics but also aging. In our study, it was observed that patients aged 65 years or older were at high risk of developing postoperative infectious complications including subhepatic abscess. Results from several past studies [8], [11], [18] are consistent with our result that advanced age was an independent risk factor. Thus, the administration of prophylactic antibiotics to elderly patients should be considered important to prevent postoperative infectious complications after laparoscopic cholecystectomy. Although the variable of bile spillage was not an independent risk factor for the overall infectious complications as shown in Table 6, and considering the higher occurrence rate of SSIs in patients with bile spillage in the No antibiotics group, prophylactic antibiotics may be more effective to prevent SSIs when a bile spillage occurs during surgery.

This study had limitations in that the methods did not involve blinding as the trial had no placebo control participants. However, there might be few biases since the doctors who checked the patients' status at the outpatient clinic were unaware of the randomization.

In summary, this trial demonstrated that antibiotic prophylaxis prevented postoperative infectious complications. The elimination of antibiotic prophylaxis did not reduce medical costs and might possibly have elevated postoperative medical costs. We conclude that perioperative administration of prophylactic antibiotics should be recommended in elective low-risk laparoscopic cholecystectomy to prevent postoperative infectious complications and to reduce medical costs.

## Supporting Information

Table S1 **All data of the trial participants.**
(XLSX)

Checklist S1 **CONSORT Checklist.**
(DOC)

IRB S1 **The approval document of the institutional review board for clinical research.**
(PDF)

Protocol S1 **Trial Protocol in Japanese.**
(PDF)

Protocol S2 **Trial Protocol in English.**
(PDF)
