# Supplementary material for: Antibiotic Prophylaxis in Laparoscopic Cholecystectomy: A Randomized Controlled Trial
Source: PLoS One. 2014 Sep 5;9(9):e106702. doi: 10.1371/journal.pone.0106702 (PMC4156368; doi:10.1371/journal.pone.0106702)
Supplement: IRB S1 — The approval document of the institutional review board for clinical research. (PDF) [file pone.0106702.s003.pdf]

平成19年3月1日

臨床研究責任者

外　科

松井陽一　殿

関西医科大学附属枚方病院

病院長　今　村　洋

院内臨床研究実施通知書

下記 臨床研究実施について、以下のとおり決定しましたので通知致します。

記

1．臨床研究名　腹腔鏡下胆嚢摘出術における抗生剤投与の必要性についての研究

受付番号　第 H070402 号

2．実施の可否　(1.)許可　2. 不許可　3. その他（　　　　）

2・3の場合その理由

3．予定症例数　1006

4．分担研究者名　里井壮平，豊川秀吉，柳本泰明

5．実施予定期間　自 平成 19 年 3 月 1 日

至 平成 23 年 4 月 30 日

Certificate of Approval

The Institutional Review Board for Clinical Research of

Kansai Medical University Hirakata Hospital

The following protocol was approved:

Title of Protocol: Antibiotic prophylaxis in laparoscopic cholecystectomy: A randomized controlled trial

Protocol Identification Number:H070402

Principal Investigator: Yoichi Matsui

Sub Investigator: Sohei Satoi, Masaki Kaibori, Hideyoshi Toyokawa, Hiroaki Yanagimoto, Kosuke Matsui, Morihiko Ishizaki, A-Hon Kwon

Period of Research: From March 1, 2007 to May 31, 2011

Name : Satoshi Sawada,M.D.

Title : Director

Kansai Medical University Hirakata Hospital

Sawada ____________________ 12/3/2014 ____________________

Signature Date

Name : Gaku Okugawa,M.D.

Title : Chair

The Institutional Review Board for Clinical Research of Kansai Medical University Hirakata Hospital

Gaku Okugawa ____________________ March 13, 2014 ____________________

Signature Date

Protocol

This protocol was registered to University Hospital Medical Information Network-Clinical Trials Registry (UMIN-CTR), registry ID: UMIN000003749

Basic information	
**Official scientific title of the study**	Role of Prophylactic Antibiotics in Laparoscopic Cholecystectomy: A Prospective Randomized Study
**Brief title**	Prophylactic antibiotics in laparoscopic cholecystectomy
**Region**	Japan

Condition	
**Condition**	Gallstone, Gallbladder polyp
**Classification by specialty**	Hepato-biliary-pancreatic surgery
**Classification by malignancy**	Benign
**Genomic information**	No

Objectives	
**Narrative objectives**	Prophylactic antibiotics are routinely used with laparoscopic cholecystectomy. However, surgical infections relating to laparoscopic cholecystectomy appear to be infrequent because this minimally invasive surgery creates only a small wound. To estimate whether prophylactic antibiotics are necessary for laparoscopic cholecystectomy, we began a randomized controlled trial.
**Basic objectives**	Safety, Efficacy
**Trial characteristics**	Exploratory, Pragmatic
**Developmental phase**	Not applicable

Assessment	
**Primary outcome**	postoperative infection-related complications
**Secondary outcomes**	postoperative hospital stay<br>postoperative medical costs

Base	
**Study type**	Interventional

Study design	
**Basic design**	Parallel
**Randomization**	Randomized
**Randomization unit**	Individual
**Blinding**	Open
**Control**	No treatment
**Stratification**	No
**Dynamic allocation**	No
**Institution consideration**	Not applicable
**Blocking**	No

Intervention	
**No. of arms**	2
**Purpose of intervention**	Prevention
**Type of intervention**	Medicine
**Intervention**	Antibiotics group: First generation cefem (1.0 g/body) is infused once intra-operatively and twice on postoperative day 1.
**Control**	No antibiotics group: No perioperative antibiotics are administered.

Eligibility	
**Age lower limit**	18
**Age upper limit**	Not applicable
**Gender**	Male and female
**Key inclusion criteria**	1. Candidate for laparoscopic cholecystectomy<br>2. No infectious disease including acute cholecystitis

	3. No severe disease such as diabetes mellitus requiring insulin, renal failure with hemodialysis, etc.<br>4. No antibiotic administration within a week before operation<br>5. Provided written informed consent
**Key exclusion criteria**	1. Switch to open surgery<br>2. Emergency surgery<br>3. Allergy to antibiotics<br>4. Obstructive jaundice
**Target sample size**	1006

**Research contact person**	
**Name of lead principal investigator**	Yoichi Matsui
**Organization**	Kansai Medical University
**Division name**	Department of Surgery
**Address**	2-3-1, Shinmachi, Hirakata city, Osaka 573-1191
**TEL**	+8172-804-0101

**Public contact**	
**Name of contact person**	Yoichi Matsui
**Organization**	Kansai Medical University, Hirakata Hospital
**Division name**	Department of Surgery
**Address**	2-3-1, Shinmachi, Hirakata city, Osaka 573-1191
**TEL**	+8172-804-0101
**Homepage URL**	https://upload.umin.ac.jp
**E-mail**	matsui@hirakata.kmu.ac.jp

**Sponsor**	
**Name of primary sponsor**	Kansai Medical University

Funding source	
**Source of funding**	Department of Surgery, Kansai Medical University
**Category of organization**	Self-funding
**IRBs**	
**Research ethics review**	Yes (approval No. H070402)
**Address**	2-3-1 Shinmachi, Hirakata city, Osaka 573-1191
**TEL**	+8172-804-0101 ext. 3465

Secondary IDs	
**Secondary IDs**	UMIN000003749

Institutions	
**Institutions**	Kansai Medical University

Plan	
**Anticipated trial start date**	2007/03/01
**Anticipated date of recruitment closure**	2011/04/30
**Anticipated last follow-up date**	2011/05/31
