# Supplementary material for: Antibiotic Prophylaxis in Laparoscopic Cholecystectomy: A Randomized Controlled Trial
Source: PLoS One. 2014 Sep 5;9(9):e106702. doi: 10.1371/journal.pone.0106702 (PMC4156368; doi:10.1371/journal.pone.0106702)
Supplement: Protocol S1 — Trial Protocol in Japanese. (PDF) [file pone.0106702.s004.pdf]

【様式１】

関 西 医 科 大 学 附 属 枚 方 病 院
臨 床 研 究 審 査 委 員 会 申 請 書

平成１９年　３月　１日

関西医科大学医学倫理委員会
附属枚方病院小委員会委員長　殿

附属病院名：　附属枚方病院
所　　属：　外科
職　　名：　助手
申 請 者 名：　松井　陽一　㊞
(研究責任者名)

受付番号（枚方病院）	※	所属長
		所属　外科　職名　主任教授<br>氏名　上山　泰男　㊞

1	課題名：	腹腔鏡下胆嚢摘出術における抗生剤投与の必要性についての研究				
2	研究責任者	所属	外科	職名	助手	松井　陽一
	分担研究者名	所属	外科	職名	助手	里井　壮平
	分担研究者名	所属	外科	職名	助手	豊川　秀吉
	分担研究者名	所属	外科	職名	助手	柳本　泰明
3	予定症例数	1006　例				
4	実施予定期間	自　2007年　3月　1日<br>至　2011年　4月　30日				
5	医薬品・検査材料入手方法	無し				

臨床研究の概要

現在、胆石症患者に対して年間約 200 例の腹腔鏡下胆嚢摘出術を行っており、全例ルーチンに術中および術後に抗生剤を点滴投与している。しかし腹腔鏡手術は、創が小さく侵襲も少ないため、術中感染の可能性がほとんどなく、実際にこれまでの数百例の経験でも術後腹腔内感染や創感染はほとんど発生していない。また抗生剤のルーチン投与は、抗生剤耐性菌の出現を助長し、さらに頻度は少ないが薬剤性肝炎や無顆粒球症など、薬剤による副作用の原因にもなり、不必要な投与は控えなければならない。そこで、胆石症患者の手術において、抗生剤投与が必要であるかどうかを調べるために、無作為化臨床試験を計画する。腹腔鏡下胆嚢摘出術において、交互に抗生剤投与群、非投与群に割付け、両群の術後経過を比較する。調査項目は、背景因子（術前の血液学的、生化学的検査所見、手術因子など）、術後の血液学的、生化学的検査所見、術後合併症、在院日数などである。症例数は各群 503 例、合計 1006 例を予定している。抗生剤投与群と非投与群で結果に差が認められなければ、腹腔鏡下胆嚢摘出術において抗生剤投与の必要性が無いと判断され、これにより抗生剤投与にかかる医療費の削減が見込まれる。現在の症例数であれば、関西医科大学枚方病院において年間約１００万円の医療費削減が可能である。世界的に見ると年間で数百億円の医療費削減が達成できる見込みである。さらに薬剤の副作用発現や耐性菌出現の抑制にもつながる。

【様式２－１－A】

関 西 医 科 大 学 附 属 枚 方 病 院
臨 床 研 究 審 査 委 員 会 申 請 書

平成１９年　３月　１日

関西医科大学附属枚方病院
病 院 長　殿

附属病院名：　附属枚方病院
所　　属：　外科
職　　名：　助手
申 請 者 名：　松井　陽一
(研究責任者名)　㊞

受付番号（枚方病院）	※	所 属 長
		所属　外科　職名　主任教授<br>氏名　上山　泰男　㊞

1	課題名：	腹腔鏡下胆嚢摘出術における抗生剤投与の必要性についての研究					
2	研究責任者	所属	外科	職名	助手	松井	陽一
	分担研究者名	所属	外科	職名	助手	里井	壮平
	分担研究者名	所属	外科	職名	助手	豊川	秀吉
	分担研究者名	所属	外科	職名	助手	柳本	泰明
3	予定症例数	1006　例					
4	実施予定期間	自　2007年　3　月　1　日<br>至　2011年　4　月　30　日					
5	医薬品・検査材料入手方法	無し					

臨床研究の概要

現在、胆石症患者に対して年間約200例の腹腔鏡下胆嚢摘出術を行っており、全例ルーチンに術中および術後に抗生剤を点滴投与している。しかし腹腔鏡手術は、創が小さく侵襲も少ないため、術中感染の可能性がほとんどなく、実際にこれまでの数百例の経験でも術後腹腔内感染や創感染はほとんど発生していない。また抗生剤のルーチン投与は、抗生剤耐性菌の出現を助長し、さらに頻度は少ないが薬剤性肝炎や無顆粒球症など、薬剤による副作用の原因にもなり、不必要な投与は控えなければならない。そこで、胆石症患者の手術において、抗生剤投与が必要であるかどうかを調べるために、無作為化臨床試験を計画する。腹腔鏡下胆嚢摘出術において、交互に抗生剤投与群、非投与群に割付け、両群の術後経過を比較する。調査項目は、背景因子（術前の血液学的、生化学的検査所見、手術因子など）、術後の血液学的、生化学的検査所見、術後合併症、在院日数などである。症例数は各群503例、合計1006例を予定している。抗生剤投与群と非投与群で結果に差が認められなければ、腹腔鏡下胆嚢摘出術において抗生剤投与の必要性が無いと判断され、これにより抗生剤投与にかかる医療費の削減が見込まれる。現在の症例数であれば、関西医科大学枚方病院において年間約１００万円の医療費削減が可能である。世界的に見ると年間で数百億円の医療費削減が達成できる見込みである。さらに薬剤の副作用発現や耐性菌出現の抑制にもつながる。

【様式２－１－B】

関西医科大学医学倫理委員会
倫　理　審　査　申　請　書

平成１９年　３月　１日

関　西　医　科　大　学
学　長　　　　　　　　殿

所　　　　属　　外科

職　　　　名　　助手
申　請　者　名　　松井　陽一
(研究責任者名)　　　　　　　　　　　　　　　　　　印

※審査区分
　A審査・B審査・C審査（医学倫理委員会委員長により記入）

受付番号	※	※は記入しないでください。	所　属　長<br>上山　泰男

1．審査を希望する委員会(どちらかに○を付けてください。)
○(1)医　学　倫　理　委　員　会
(2)ヒトゲノム・遺伝子解析研究倫理審査小委員会
(3)ヒトＥＳ細胞樹立・使用研究倫理審査小委員会
(4)遺伝子治療臨床研究審査小委員会
(5)疫学研究倫理審査小委員会

2．課題名

腹腔鏡下胆嚢摘出術における抗生剤投与の必要性についての研究

3. 研究責任者	所属	外科	職名	助手	松井　陽一
分担研究者名	所属	外科	職名	助手	里井　壮平
分担研究者名	所属	外科	職名	助手	豊川　秀吉
分担研究者名	所属	外科	職名	助手	柳本　泰明

4．個人情報管理者(ヒトゲノム・遺伝子解析研究倫理審査小委員会で審査を希望する場合のみ記入。)

個人情報管理者名(必須)：　　　　所　属：　　　　職　名：

分担管理者名（任意)：　　　　所　属：　　　　職　名：

5．研究等の概要

現在、胆石症患者に対して年間約200例の腹腔鏡下胆嚢摘出術を行っており、全例ルーチンに術中および術後に抗生剤を点滴投与している。しかし腹腔鏡手術は、創が小さく侵襲も少ないため、術中感染の可能性がほとんどなく、実際にこれまでの数百例の経験でも術後腹腔内感染や創感染はほとんど発生していない。また抗生剤のルーチン投与は、抗生剤耐性菌の出現を助長し、さらに頻度は少ないが薬剤性肝炎や無顆粒球症など、薬剤による副作用の原因にもなり、不必要な投与は控えなければならない。そこで、胆石症患者の手術において、抗生剤投与が必要であるかどうかを調べるために、無作為化臨床試験を計画する。腹腔鏡下胆嚢摘出術において、交互に抗生剤投与群、非投与群に割付け、両群の術後経過を比較する。調査項目は、背景因子（術前の血液学的、生化学的検査所見、手術因子

など)、術後の血液学的、生化学的検査所見、術後合併症、在院日数などである。症例数は各群 503 例、合計 1006 例を予定している。抗生剤投与群と非投与群で結果に差が認められなければ、腹腔鏡下胆嚢摘出術において抗生剤投与の必要性が無いと判断され、これにより抗生剤投与にかかる医療費の削減が見込まれる。現在の症例数であれば、関西医科大学枚方病院において年間約１００万円の医療費削減が可能である。世界的に見ると年間で数百億円の医療費削減が達成できる見込みである。さらに薬剤の副作用発現や耐性菌出現の抑制にもつながる。

６．研究等における医学倫理的、社会的配慮について

Ⅰ．研究等の対象となる個人の人権の擁護

試験への参加は被験者の自由意志によるものであり、被験者は試験への参加を随時拒否または撤回できる。拒否や撤回により被験者が不利な扱いを受けることはない。また、試験に参加しない場合でも受けるべき利益は失われないことを説明文書で保証する。

登録患者の個人情報は記号化し、主任研究者を除き被験者個人を特定できないようにする。個人情報は管理者を主任研究者のみに限定し、分担研究者の閲覧も禁止し、部外者へ漏えいする危険性を極力排除する。この試験に関する個人情報を当施設から他施設や個人へ通知することはない。個々の被験者は、試験計画及び方法についての情報を、他の被験者の個人情報保護に支障のない範囲で入手、閲覧が可能である。試験の結果が公表される場合、被験者の個人情報は完全に保全される。

Ⅱ．研究等の対象となる者に理解を求め同意を得る方法

被験者に添付の説明文書を用いて、本研究により被験者に及び得る利害得失、並びに医学、医療への期待される貢献について説明する。試験への参加は被験者の自由意志によるものであり、被験者は試験への参加を随時拒否または撤回できる。拒否や撤回により被験者が不利な扱いを受けることはない。また、試験に参加しない場合でも受けるべき利益は失われない。これらに関して被験者に口頭及び文書で説明し、十分に理解をしていただいた後に、同意を得られた場合には添付の同意書に署名をしてもらう。

Ⅲ．研究等によって生じる個人への不利益並びに危険性に対する配慮

この研究に参加した場合の予測される不利益並びに危険性は、抗生剤非投与群において術後感染性合併症の頻度が高まることである。これに対処するために、まず個々の症例においては非投与群において感染性合併症の兆候が認められた場合、直ちに抗生剤投与を含む治療を開始する。さらに試験開始後、両群合わせた症例数が50例毎に統計学的評価を行い、期待される結果と異なる統計学的判定、すなわち抗生剤非投与群において有害事象発生頻度が有意に高い場合はこの臨床試験を中止する。有意差が認められなければ予定症例数まで試験を継続する。また逆に、抗生剤投与群の有害事象発生頻度が有意に高い場合でも、現時点では予防的抗生剤投与が一般的に標準治療と考えられるため、予定症例数まで試験を継続する。しかし明らかに差が認められ、有害事象が無視できない場合にはこの限りではなく、その時点で試験継続につき再検討する。

これら有害事象発生時には、所属長、研究責任者、分担研究者の全員で、有害事象が発生した個々の患者の対応及び研究継続の検討を行う。

Ⅳ．医学並びに社会への貢献度の予測

これまでの臨床経験よりみて、胆嚢炎のない待機的手術のみでなく、急性胆嚢炎に対する緊急手術であっても、腹腔鏡下胆嚢摘出術後に腹膜炎や創感染の合併症はほとんど発生していない。数百例の腹腔鏡下胆嚢摘出手術に対し、外科的処置を必要とする術後の腹腔内感染、創感染は皆無であった。それに対して、術後抗生剤による薬剤性肝炎を疑われる症例が数例認められており、抗生剤投与がかえって患者に有害である可能性も考えられる。これらのことより、この臨床試験により抗生剤投与の有用性が否定されれば、無駄な医療費の削減に加えて、患者に与える有害事象の抑制に貢献でき、さらには抗生剤耐性菌の抑制にもつながるものと思われる。

Ⅴ．その他

【様式２－３】

研究等実施計画書

1　課題名

腹腔鏡下胆嚢摘出術における抗生剤投与の必要性についての研究

2　目　的

胆石症患者の手術において、術中術後の抗生剤投与が必要であるかどうかを調べることを目的とする。

3　対　象

胆嚢摘出術を必要とする患者で以下の基準を満たすものとする。

選択基準：

1. 18 歳以上の腹腔鏡下胆嚢摘出術の予定手術患者
2. 胆嚢炎を含む細菌感染症を合併していない
3. 免疫不全、透析を必要とする腎不全、インスリンを必要とする糖尿病など、重得な基礎疾患を合併していない
4. 術前 1 週間以内に抗生剤を投与されていない
5. 文書で同意が得られる

除外基準：

1. 腹腔鏡手術から開腹手術に移行した場合
2. 緊急手術の場合
3. 閉塞性黄疸を合併している場合
4. 抗生剤に対しアレルギーがある場合
5. 認知症など判断能力がないと判断される場合

（術前診断は問わない。）

4　方　法

無作為化臨床試験：

腹腔鏡下胆嚢摘出術の手術患者を、手術の順番に交互に術中術後抗生剤投与を行う群と行わない群に割り付ける。抗生剤投与群は術中から術翌日までの２日間、第一世代セフェム系抗生剤を点滴投与する。非投与群は経口、点滴を含め入院期間中に抗生剤投与を行わない。術後の合併症発症率、在院日数、血液検査所見などを評価する。評価期間は術後から退院までとする。

評価項目：

1. Primary end point：創感染、腹腔内感染など局所的な感染症と全身感染症の術後合併症の有無
2. 血液検査所見：術前後の白血球数、CRP、T. Bil、D. Bil、AST、ALT、ALP、GGT、AMY、BUN、Cr
3. 手術関連因子：手術時間、術中胆汁の漏れの有無、術中造影の有無、洗浄液量、ドレインの有無、
4. 胆嚢標本の肉眼的及び病理学的所見
5. 術前後の体温
6. 術後在院日数

5　実施場所

関西医科大学枚方病院

平成１９年　３月　１日

申請者(研究責任者名) 名　　　松井　陽一　　印

予防的抗生剤投与の必要性を検証する臨床試験についての説明書

現在当院では、胆石症の患者様に対して年間約１５０例の腹腔鏡下胆嚢摘出術を行っており、全例に手術中および術後に細菌感染を予防する目的で抗生剤を点滴投与しています。しかし腹腔鏡手術は創が小さく、身体に与えるストレスが少ないため、術後細菌感染の可能性がほとんどないと思われます。また抗生剤の投与は抗生剤耐性菌の出現を増やし、さらに薬剤性肝炎や無顆粒球症、アレルギーによる皮膚炎やショックなどの原因にもなり、不必要な抗生剤投与は控えなければなりません。

これまでのわれわれの臨床経験よりみて、胆嚢炎のない予定手術のみでなく、胆嚢に細菌感染のある急性胆嚢炎に対する緊急手術であっても、腹腔鏡下胆嚢摘出術後に腹膜炎や創感染の合併症はほとんど発生していません。数百例の腹腔鏡下胆嚢摘出手術に対し、なんらかの処置を必要とする術後の腹腔内の細菌感染や創の感染はほとんどありませんでした。それに対して、術後に抗生剤による薬剤性肝炎を疑われた症例が数例認められており、抗生剤投与がかえって患者様に有害である可能性も考えられます。

そこで胆石症の患者様の手術において、このような抗生剤投与が本当に必要であるかどうかを調べるために臨床試験を行っています。胆嚢摘出術において抗生剤投与をする患者様と、しない患者様に順番に振り分け、それぞれの患者様の術後経過を比較しています。調査内容は、手術前の血液検査、手術内容などと、手術後の血液検査、術後合併症の有無、入院日数などです。臨床試験の症例数は合計５００例を予定しています。

抗生剤投与のありなしで結果に差がなければ、胆嚢摘出術において予防的な抗生剤投与の必要性が無いということになります。これにより抗生剤投与にかかる医療費の削減が見込まれます。現在の症例数であれば、関西医科大学枚方病院において年間約１００万円の医療費削減が可能であり、世界規模で見ると年間でおおよそ数百億円の医療費削減が達成できる見込みです。また無駄な医療費の削減に加えて、患者様に与える抗生剤の有害な副作用の抑制にも貢献できるものと思われます。さらに抗生剤の効かない薬剤耐性菌出現の抑制にもつながります。

この研究に参加することで抗生剤を投与しない群に選ばれた場合、メリットとして抗生剤による副作用（薬剤性肝炎、無顆粒球症、アレルギー性皮膚炎、アナフィラキシーショックなどの薬害）の発生がなくなるという点です。デメリットとしては術後細菌感染症（腹腔内感染や創感染など）の発生する率が高まるかもしれないという点です。また抗生剤を投与する群に選ばれた場合、メリットは術後細菌感染症が予防できる可能性があるという点です。デメリットは抗生剤による薬害の可能性があるという点です。

研究に参加したことによる有害事象（具体的には、抗生剤を投与しなかった場合の術後細菌性感染症）が発症あるいはその兆候が認められた場合、直ちに抗生剤投与を含む治療を開始します。

この研究に参加するかしないかはご本人の自由です。そして参加する、しないに関わらず医療上何ら不利益や不都合が生じることはありません。参加後にいつでも自由に参加を中止することができます。また参加する事に対しての費用はかかりません。

この研究に関して何か疑問に思われる点がありましたらどうぞお気軽にご連絡下さい。

担当医　　松井　陽一

〒573-1191 大阪府枚方市新町 2-3-1
関西医科大学　外科　權　雅憲
Tel: 072-804-0101, 内線 56128

予防的抗生剤投与の必要性を検証する臨床試験参加の同意書

私は、関西医科大学　外科　担当医　松井陽一　よりこの臨床研究に関する説明を受け、それを理解し、納得した上で、この同意はいつでも撤回できることを条件として、この研究に参加することに同意します。

______年______月______日　住所 ________________________

氏名 ________________________
