# Supplementary material for: Antibiotic Prophylaxis in Laparoscopic Cholecystectomy: A Randomized Controlled Trial
Source: PLoS One. 2014 Sep 5;9(9):e106702. doi: 10.1371/journal.pone.0106702 (PMC4156368; doi:10.1371/journal.pone.0106702)
Supplement: Protocol S2 — Trial Protocol in English. (PDF) [file pone.0106702.s005.pdf]

UMIN CTR 臨床試験登録情報の閲覧

利用者名：松井 陽一 UMIN ID：ymatsui-kmu

試験進捗状況	：試験終了/Completed
ＵＭＩＮ試験ＩＤ	：UMIN000003749
試験名	：腹腔鏡下胆嚢摘出術における抗生剤投与の必要性についての研究
登録日（＝情報公開日）	：2010/06/14
最終データ内容更新日時	：2013/08/07 14:55:42

※ 本ページ収載の情報は、臨床試験に関する情報公開を目的として、UMINが開設しているUMIN臨床試験登録システムに提供された臨床試験情報です。

※ 特定の医薬品や治療法等については、医療関係者や一般の方に向けて広告することは目的としていません。

基本情報 (Basic information)		
項目(Item)	日本語(Japanese)	英語(English)
試験名 (Official scientific title of the study)	腹腔鏡下胆嚢摘出術における抗生剤投与の必要性についての研究	Role of Prophylactic Antibiotics in Laparoscopic Cholecystectomy: A Prospective Randomized Study
試験簡略名 (Title of the study (Brief title))	腹腔鏡下胆嚢摘出術における抗生剤投与の必要性	Prophylactic antibiotics in laparoscopic cholecystectomy
試験実施地域 (Region)	日本/Japan	

対象疾患(Condition)		
項目(Item)	日本語(Japanese)	英語(English)
対象疾患名 (Condition)	胆石症、胆嚢ポリープ	Gallstone, Gallbladder polyp
疾患区分1 (Classification by specialty)	消化器外科（肝・胆・膵）/Hepato-biliary-pancreatic surgery	

疾患区分2 (Classification by malignancy)	悪性腫瘍以外/Others
ゲノム情報の取扱い (Genomic information)	いいえ/NO

目的(Objectives)		
項目(Item)	日本語(Japanese)	英語(English)
目的1 (Narrative objectives1)	外科手術においては一般的に術後感染に対する予防的抗生剤投与が行なわれているが、腹腔鏡下手術では侵襲が少なく術後感染症が起こりにくいと考えられている。最近、待機的腹腔鏡下胆嚢摘出術における予防的抗生剤投与の必要性を検討した無作為化試験が多数報告され、それらのメタアナリシスもいくつか報告されている。それらの結果はいずれも待機的腹腔鏡下胆嚢摘出術における予防的抗生剤投与を不要とする結果であった。しかし報告された各臨床試験は症例数が限られている。今回我々はその検証を行なうため無作為化臨床試験を行った。	Prophylactic antibiotics are routinely used with laparoscopic cholecystectomy. However, surgical infections relating to laparoscopic cholecystectomy appear to be infrequent because this minimally invasive surgery creates only a small wound. To estimate whether prophylactic antibiotics are necessary for laparoscopic cholecystectomy, we began a randomized controlled trial.
目的2 (Basic objectives2)	安全性・有効性/Safety,Efficacy	
目的2 -その他詳細 (Basic objectives - Others)		
試験の性質1 (Trial characteristics 1)	探索的/Exploratory	
試験の性質2 (Trial characteristics 2)	実務的/Pragmatic	
試験のフェーズ (Developmental phase)	該当せず/Not applicable	

評価 (Assessment)

項目(Item)	日本語(Japanese)	英語(English)
主要アウトカム評価項目 (Primary outcomes)	術後感染性合併症	postoperative infection-related complications
副次アウトカム評価項目 (Key secondary outcomes)	術後在院日数<br>術後医療費	postoperative hospital stay<br>postoperative medical costs

基本事項 (Base)		
項目(Item)	日本語(Japanese)	英語(English)
試験の種類 (Study type)	介入/Interventional	

試験デザイン (Study design)		
項目(Item)	日本語(Japanese)	英語(English)
基本デザイン (Basic design)	並行群間比較/Parallel	
ランダム化 (Randomization)	ランダム化/Randomized	
ランダム化の単位 (Randomization unit)	個別/Individual	
ブラインド化 (Blinding)	試験参加者がブラインド化されている単盲検/Single blind -participants are blinded	
コントロール (Control)	無治療対照/No treatment	
層別化 (Stratification)	いいえ/NO	
動的割付 (Dynamic allocation)	いいえ/NO	
試験実施施設の考慮 (Institution consideration)	施設を考慮していない/Institution is not considered as adjustment factor.	

ブロック化 (Blocking)	いいえ/NO
割付コードを知る方法 (Concealment)	封筒法/Numbered container method

介入 (Intervention)		
項目(Item)	日本語(Japanese)	英語(English)
群数 (No. of arms)	2	
介入の目的 (Purpose of intervention)	予防・検診・検査/Prevention	
介入の種類 (Type of intervention)	医薬品/Medicine	
介入1 (Interventions/Control_1)	対照群は手術直前と術翌日に2回、第1世代セフェム系抗生剤をそれぞれ1g点滴投与。	Antibiotic group: First generation cefem (1.0 g/body) is infused once intraoperatively and twice on postoperative day 1.
介入2 (Interventions/Control_2)	介入群は抗生剤をいっさい投与しない。	No antibiotic group: No perioperative antibiotics are administered.
介入3 (Interventions/Control_3)		
介入4 (Interventions/Control_4)		
介入5 (Interventions/Control_5)		
介入6 (Interventions/Control_6)		
介入7 (Interventions/Control_7)		
介入8 (Interventions/Control_8)		
介入9 (Interventions/Control_9)		
介入10		

(Interventions/Control_10)		

適格性 (Eligibility)		
項目(Item)	日本語(Japanese)	英語(English)
年齢（下限） (Age-lower limit)	18 歳/years-old 以上/<=	
年齢（上限） (Age-upper limit)	適用なし/Not applicable	
性別 (Gender)	男女両方/Male and Female	
選択基準 (Key inclusion criteria)	1. 待機的腹腔鏡下胆嚢摘出術<br>2. 感染症がない<br>3. インスリンを要する糖尿病、透析などの重症疾患がない<br>4. 手術 1 週間以内に抗生剤を投与されていない<br>5. 書面で同意が得られる	1. Candidate for laparoscopic cholecystectomy<br>2. No infectious disease including acute cholecystitis<br>3. No severe disease such as diabetes mellitus requiring insulin, renal failure with hemodialysis, etc.<br>4. No antibiotic administration within a week before operation<br>5. Provided written informed consent
除外基準 (Key exclusion criteria)	1. 開腹移行<br>2. 緊急手術<br>3. 抗生剤アレルギーの既往歴<br>4. 閉塞性黄疸	1. Switch to open surgery<br>2. Emergency surgery<br>3. Allergy to antibiotics<br>4. Obstructive jaundice
目標参加者数 (Target sample size)	1000	

責任研究者 (Research contact person)		
項目(Item)	日本語(Japanese)	英語(English)
責任研究者名 (Name of lead principal investigator)	松井陽一	Yoichi Matsui
所属組織 (Organization)	関西医科大学	Kansai Medical University
所属部署 (Division name)	外科学講座	Department of Surgery

住所 (Address)	大阪府枚方市新町2-3-1	2-3-1 Shinmachi Hirakata Osaka
電話 (TEL)	0728040101	

試験問い合わせ窓口(Public contact)		
項目(Item)	日本語(Japanese)	英語(English)
担当者名 (Name of contact person)	松井陽一	Yoichi Matsui
組織名 (Organization)	関西医科大学	Kansai Medical University
部署名 (Division name)	外科学講座	Department of Surgery
住所 (Address)	大阪府枚方市新町2-3-1	2-3-1 Shinmachi Hirakata Osaka
電話 (TEL)	0728040101	
試験のホームページURL (Homepage URL)		
Email (Email)	matsui@hirakata.kmu.ac.jp	

情報送信組織 (Organization sending information)		
項目(Item)	日本語(Japanese)	英語(English)
送信者名 (Name of person sending information)	松井 陽一	Yoichi Matsui
情報送信組織 (Organization)	関西医科大学（大学院）	Kansai Medical University
所属部署 (Division name)	外科学講座	Department of Surgery
住所	大阪府枚方市新町2丁目3番1号関西医科大	Hirakata Hospital, Kansai Medical University,

(Address)	学附属枚方病院	2-3-1 Shinmachi, Hirakata, Osaka
電話 (TEL)	072-804-0101	
Email (Email)	matsui@hirakata.kmu.ac.jp	

実施責任組織 (Sponsor)		
項目(Item)	日本語(Japanese)	英語(English)
実施責任組織 (Name of primary sponsor)	関西医科大学外科学講座	Department of Surgery Kansai Medical University

実施責任組織とは、「試験の計画、解析と結果公表、研究費調達を含めた実施のための運営管理に対して責任を持つ組織」です。英語名でスポンサーとありますが、通常イメージする資金提供者のことではございません。従いまして、「なし」という記載はありえません。

研究費提供組織(Funding Source)		
項目(Item)	日本語(Japanese)	英語(English)
研究費提供組織 (Source of funding)	関西医科大学外科学講座	Department of Surgery Kansai Medical University
組織の区分 (Category of Org.)	自己調達/Self funding	
研究費拠出国 (Nation of funding)		

その他の関連組織 (Other related organizations)		
項目(Item)	日本語(Japanese)	英語(English)
共同実施組織 (Name of secondary sponsor(s))		

その他の研究費提供組織 (Name of secondary fun d er(s))		

IRB等連絡先 (IRBs)		
項目(Item)	日本語(Japanese)	英語(English)
倫理委員会による審査・承認 (Research ethics review)	あり/YES	
日本の法規に定める医薬品製造業者等による医薬品の市販後調査への該当 (Post marketing survey by drug manufacture etc., specified by Japanese law.)	該当なし（医薬品製造業者等でない場合は、こちらを選択ください）/Not applicable (Chose this category if you are not drug manufacture etc. in Japan)	
組織名１ (Organization1)	関西医科大学	
住所１ (Address1)	大阪府枚方市新町2-3-1	
電話１ (Tel1)	0728040101ext.3465	
Email1 (Email1)	matsui@hirakata.kmu.ac.jp	
組織名2 (Organization2)		
住所2 (Address2)		
電話２ (Tel2)		
Email2 (Email2)		
組織名3 (Organization3)		

住所3 (Address3)	
電話３ (Tel3)	
Email3 (Email3)	

他機関から発行された試験ID (Secondary IDs)		
項目(Item)	日本語(Japanese)	英語(English)
他機関から発行された試験ＩＤ (Secondary IDs)	いいえ/NO	
試験ID1 (Study ID 1)		
ID発行機関1 (Org. issuing International ID 1)		
試験ID2 (Study ID 2)		
ID発行機関2 (Org. issuing International ID 2)		
治験届 (IND to MHLW)		

試験実施施設 (Institutions)		
項目(Item)	日本語(Japanese)	英語(English)
試験実施施設名称 (Institutions)	関西医科大学附属枚方病院	

その他の管理情報 (Other administrative information)		
項目(Item)	日本語(Japanese)	英語(English)
試験情報の本登録希		

望日 (Date of disclosure of the study information)	2010/06/14

試験進捗状況 (Progress)		
項目(Item)	日本語(Japanese)	英語(English)
試験進捗状況 (Recruitment status)	試験終了/Completed	
プロトコル確定日 (Date of protocol fixation)	2007/03/10	
登録・組入れ開始（予定）日 (Anticipated trial start date)	2007/03/01	
フォロー終了(予定)日 (Last follow-up date)	2013/06/30	
入力終了(予定)日 (Date of closure to data entry)	2013/06/30	
データ固定（予定）日 (Date trial data considered complete)	2013/06/30	
解析終了(予定)日 (Date analysis concluded)	2013/07/31	

関連情報 (Related information)		
項目(Item)	日本語(Japanese)	英語(English)
プロトコル掲載URL (URL releasing		

protocol)		
試験結果の公開状況 (Publication of results)	未公表/Unpublished	
結果掲載URL (URL releasing results)		
主な結果 (Results)		
その他関連情報 (Other related information)		

更新許可者（UMIN user permited to amend)		
項目(Item)	日本語(Japanese)	英語(English)
更新許可者 UMIN ID1 (UMIN ID1)		
更新許可者 UMIN ID2 (UMIN ID2)		
更新許可者 UMIN ID3 (UMIN ID3)		

管理情報		
項目(Item)	日本語(Japanese)	英語(English)
UMIN試験ID (Unique ID issued by UMIN)	UMIN000003749	
状態 (Status)	登録済み/Regist	
初回申請者 (Applicant for provisional registration)	ymatsui-kmu (松井 陽一)	
申請日		

(Date of provisional registration)	2010/06/11 16:38:52
登録者 (Applicant for registration)	ymatsui-kmu (松井 陽一)
登録日 (Date of registration)	2010/06/14
更新者 (Person last updated)	ymatsui-kmu (松井 陽一)
最終情報更新日 (Date of last update)	2013/08/07 14:55:42

更新 戻る

UMIN臨床試験登録システムのご使用に関するお問い合わせは、こちらのお問い合わせフォーム からお願いいたします。それ以外のお問い合わせは、こちら よりお願い致します。

UMIN Infrastructure for Academic Activities
University hospital Medical Information Network
